# Does Sleep Improve Your Grammar? Preferential Consolidation of Arbitrary Components of New Linguistic Knowledge

**DOI:** 10.1371/journal.pone.0152489

**Published:** 2016-04-05

**Authors:** Jelena Mirković, M. Gareth Gaskell

**Affiliations:** 1 Faculty of Health and Life Sciences, York St John University, Lord Mayor's Walk, York, YO31 7EX, United Kingdom; 2 Department of Psychology, University of York, York, YO10 5DD, United Kingdom; University of Barcelona, SPAIN

## Abstract

We examined the role of sleep-related memory consolidation processes in learning new form-meaning mappings. Specifically, we examined a Complementary Learning Systems account, which implies that sleep-related consolidation should be more beneficial for new hippocampally dependent arbitrary mappings (e.g. new vocabulary items) relative to new systematic mappings (e.g. grammatical regularities), which can be better encoded neocortically. The hypothesis was tested using a novel language with an artificial grammatical gender system. Stem-referent mappings implemented arbitrary aspects of the new language, and determiner/suffix+natural gender mappings implemented systematic aspects (e.g. ***tib*** *scoiff****esh*** *+* ballerina, ***tib*** *mof****eem*** + bride; ***ked*** *jor****ool*** + cowboy, ***ked*** *heef****aff*** + priest). Importantly, the determiner-gender and the suffix-gender mappings varied in complexity and salience, thus providing a range of opportunities to detect beneficial effects of sleep for this type of mapping. Participants were trained on the new language using a word-picture matching task, and were tested after a 2-hour delay which included sleep or wakefulness. Participants in the sleep group outperformed participants in the wake group on tests assessing memory for the arbitrary aspects of the new mappings (individual vocabulary items), whereas we saw no evidence of a sleep benefit in any of the tests assessing memory for the systematic aspects of the new mappings: Participants in both groups extracted the salient determiner-natural gender mapping, but not the more complex suffix-natural gender mapping. The data support the predictions of the complementary systems account and highlight the importance of the arbitrariness/systematicity dimension in the consolidation process for declarative memories.

## Introduction

Sleep-related memory consolidation processes play an important role in language learning [1–5]. For example, in novel word learning sleep-related consolidation both strengthens the newly acquired phonological forms, and helps the integration of the new forms with the existing lexicon [1, 3, 4]. The transfer of information from the hippocampus to the neocortex occurring during sleep is assumed to underlie these processes [4]. In infants, sleep has been shown to benefit the learning of word meanings [6], as well as the abstraction of statistical regularities in a string of nonsense words, a process thought to help grammar learning [2, 7].

The majority of the studies investigating the role of sleep in language learning have focused on the word’s phonological form [1–4]. However, word knowledge includes many other types of information, such as what the word means or what its grammatical properties are [8–10]. The mapping between a word’s form (spoken, written) and its meaning underpins these aspects of word knowledge. Crucially, the level of systematicity of this mapping varies such that for most words the relationship between a word’s phonological form and its meaning is highly arbitrary. For example, although the words *apple* and *cherry* are similar in meaning (e.g. they are both fruit), they do not share any similarities in the form. However, grammatical properties of words rely strongly on the systematicity of the sound-meaning mapping [11]. For example, in English nouns there is a high degree of systematicity in the mapping between the plural morpheme–*s* and its meaning (more than one object, e.g. *apples*, *cherries*). This type of variation in systematicy/arbitrariness in the sound to meaning mapping is thought to serve distinct purposes in language learning and use, with systematicity facilitating category learning and arbitrariness meaning individuation [11] (note also the distinction between the notion of systematicity as discussed here and other type of regularities in the form-meaning mapping as in onomatopoeias [12]. In the current study we use the variation in the systematicity/arbitrariness of the sound-meaning mapping to further explore the role of sleep-related consolidation in language learning. Specifically, we investigate the involvement of sleep-related consolidation in strengthening arbitrary mappings, as well as in extracting systematic regularities that exist across them.

More broadly, sleep is understood to play an important role in consolidation of memory, with evidence that it can stabilize and strengthen individual memories [13–15], as well as help the extraction of regularities across individual memories [16]. The dominant account of these processes draws on longstanding models of systems consolidation [17–20], in which memories are supported by two systems, a hippocampal and a neocortical system. The hippocampal system is central to the encoding of memories, whereas the neocortex provides a long-term basis for consolidated memories. The role of sleep by this account is to support the cross-talk between the two systems to allow hippocampal memories to become embedded neocortically over time [14, 21]. This notion is consistent with evidence for hippocampal replay during sleep from both animal and human studies [15, 22–24].

The Complementary Learning Systems theory [25] extends systems consolidation by supplementing the neuroscientific description of systems consolidation with a more explicit computational grounding. By this account the hippocampal system is assumed to encode information in a way that strengthens pattern separation [26], and thus this system is particularly important for the initial encoding of arbitrary mappings [25, 27]. At the same time, weak learning is possible independently of the hippocampus within the neocortex [28], which uses distributed and highly overlapping representations [25]. This cortical encoding scheme is particularly beneficial for initial learning of systematic mappings in that the highly overlapping representations that would build up across many consistent training episodes would lead to more reliable neocortical representation without the need for reliance on the hippocampus [25, 29].

Although the computational division of labor in the complementary systems account is well understood, its predictions have never been fully fleshed out with respect to the involvement of sleep (although cf. [30]). However, a simple marriage of our current understanding of sleep-associated consolidation with complementary systems theory leads to the prediction that if arbitrary mappings are more strongly reliant on hippocampal learning, then these mappings should also show greater effects of sleep through hippocampal replay [22–24]. In the current study we focus not on the neural circuitry underlying the complementary systems account but instead on the specific behavioral predictions that derive from these computational principles. In particular, we test the above hypothesis by investigating the extent to which the contribution of sleep-related consolidation varies with the systematicity in the newly learned mappings. Specifically, sleep-related consolidation of the arbitrary mappings may result in the strengthening of individual memories. However, sleep-related consolidation processes may play little or no role in the consolidation of systematic mappings, which can be better supported by the neocortical system at acquisition via gradual extraction of the regularities across multiple training examples.

We investigated this issue using an artificial language designed to incorporate both arbitrary and systematic mappings within the same stimulus set in a similar way as it is found in natural languages [11, 12]. To achieve this goal, the artificial language was designed to mimic grammatical gender systems. Grammatical gender systems in natural languages typically have a major semantic dimension (e.g. natural gender or animacy of the referent) as a semantic core [31]. For example, in many Indo-European languages words for referents with different natural genders have different grammatical genders too (e.g. in Spanish *vaca*_feminine_—cow and *toro*_masculine_−bull [the subscript denotes the grammatical gender of the word]). Semantic properties associated with different grammatical genders often extend beyond the natural sex of the referent, or its animacy, and include other, more subtle semantic features [31–33]. Grammatical gender in natural languages is marked by the word’s phonological properties, for instance its ending (e.g. in Spanish: *vac****a***_feminine_−cow, *tor****o***_masculine_−bull; *chic****a***_feminine_−girl, *chic****o***_masculine_−boy; *actr****iz***_feminine_−actress, *act****or***_masculine_—actor). Moreover, words of different grammatical genders are additionally marked in other elements in the sentence, for example a preceding determiner (e.g. in Spanish ***la*** *vaca*_feminine_, ***la*** *chica*_feminine_, ***la*** *actriz*_feminine_; ***el*** *toro*_masculine_, ***el*** *chico*_masculine_, ***el*** *actor*_masculine_). This redundancy of the cues to a grammatical property is common to many grammatical systems in human language [34].

In psycholinguistics, the learning of meanings (i.e. arbitrary form-meaning mappings) and the learning of grammar have traditionally been considered separate learning problems requiring different learning mechanisms [35]. However, more recent research points to the similarities in these two learning problems (see, for example [36]), in particular with regards to both involving tracking statistical regularities in the input [37–40]. For example, several studies have demonstrated the role of cross-situational word-referent co-occurrence for the learning of word meanings [40–43]. In grammar learning, children and adults use probabilistic phonological cues associated with different grammatical categories [37, 44–46]. These cues are also used in online processing [47, 48]. In visual word recognition, several studies have demonstrated the role of form-meaning [49–52] and orthographic regularities (see [53] for a review) in the processing of morphologically complex words. Together, this research suggests that statistical regularities in the form to meaning mapping are used in both word and grammar learning. While this does not rule out the existence of additional learning mechanisms in language learning [35, 54], the current study focuses on what these two learning problems have in common and how the learning of arbitrary and systematic aspects of the mappings are influenced by sleep-related memory consolidation processes.

We employed the notion of grammatical gender to design an artificial language that had varying levels of systematicity in the sound-meaning mapping. Each phrase in the language contained two words (a determiner followed by a noun) referring to a picture. For example, the sequence *tib scoiffesh* might be used to refer to a picture of a ballerina. In this example, *tib* has the grammatical function of a determiner and *scoiffesh* is composed of a stem (*scoiff-*) and a suffix (*-esh*). The stems (e.g., *scoiff-*, *jor-*) were low in systematicity, in that they had an entirely random (arbitrary) relationship with their associated meanings (e.g., ballerina, cowboy). These components of the language should rely heavily on sparse encoding/hippocampal mediation in initial learning according to a complementary systems account, because there are no shared aspects across different instances of the mapping. For example, learning that *scoiff-* refers to ‘ballerina’ does not help the learner to predict that *jor*- refers to ‘cowboy’.

We implemented the more systematic aspect of the mapping between form and meaning using two types of cues modeled on grammatical gender systems. (Note that grammatical gender systems in natural languages contain a much greater degree of complexity than the artificial language employed in the current study, but in common with many studies (see e.g. [55] for a review) we focus here on key aspects such as systematic and arbitrary components rather than a fully fledged system.) In the current study the natural gender of the referents (i.e. their sex as depicted) served as the semantic core of the artificial language, such that the novel words for female and male referents (e.g. different occupations that have female or male stereotypes associated with them) were designated as grammatically feminine and masculine respectively. The form cues indicating grammatical gender were the determiners (*tib* or *ked*) and the suffixes attached to the stems (*-esh*, *-eem*, *-ool*, *-aff*). Each determiner was consistently presented with words of a particular gender (e.g. *tib* with feminine, *ked* with masculine: *tib scoiffesh*–ballerina, *ked jorool*–cowboy). The word endings provided a second, redundant cue to gender, but instead of the one-to-one mapping between the determiner and the gender, there were two suffixes associated with each gender (i.e.,–*esh*, *-eem* for feminine: *tib scoiff****esh—***ballerina, *tib mof****eem—***bride; *-ool*, *-aff* for masculine: *ked jor****ool—***cowboy, *ked heef****aff***—priest). The use of two redundant cues (a determiner and a suffix) provides a better approximation to real gender systems in human languages [31]. In addition, the use of two cues was likely to lead to a broader spread of performance levels given that the type of cue implemented in the suffixes is often found more difficult in second language acquisition due to their lower salience and cue redundancy [56, 57]. A wider range of performance in the systematic aspects of the mapping would give us a better opportunity to pick up any beneficial effects of sleep on the consolidation of this knowledge.

The artificial language used in the current study captures an arbitrary component in individual stem to picture mappings (e.g. *scoiff*–ballerina, *jor*–cowboy), and the systematic components in the mapping between determiners/suffixes and a semantic feature common to a set of items (e.g. *tib…esh/eem* and female; *ked…ool/aff* and male). The systematic mappings can be generalized to untrained exemplars, similar to the generalization occurring with grammatical morphemes in natural languages (e.g. the wugs test [58]). As in morphological systems in natural languages, which are a ubiquitous form of non-arbitrariness in human language, the systematicity in the current study is reflected in the statistical regularities that apply to a group of words (see [12].for further discussion).

We investigated the acquisition and consolidation of the artificial language incorporating arbitrary and systematic mappings in participants who either slept or stayed awake after learning. The extraction of the more systematic aspects of the mappings (determiners and suffixes) was assessed both in the trained items and, particularly, in the ability to generalize any acquired knowledge to a set of new untrained picture-word pairs. Whereas there is ambiguity regarding the extent to which the memory of the systematic aspects of the trained pairs relies on individual item knowledge, the interpretation is clearer for the tests involving generalization to new previously unseen pairs.

We used a nap paradigm to assess the contribution of sleep-related consolidation, given previous evidence that short daytime naps produce similar effects to overnight sleep in terms of both strengthening and stabilization of newly acquired memories [59–62], and in the extraction of regularities [2, 7, 63–65]. We predicted that there would be a dissociation in the benefit of the nap to the acquisition of the new language based on the systematicity of the different components of the form-meaning mapping. More systematic aspects of the mapping (grammatical gender knowledge) should be better learned via distributed neocortical networks, and so should be relatively unaffected by time spent awake versus asleep. On the other hand, more arbitrary aspects of the mapping (vocabulary knowledge) should be acquired principally via hippocampal mediation and so should show a greater benefit of a nap over time spent awake.

**Fig 1:**
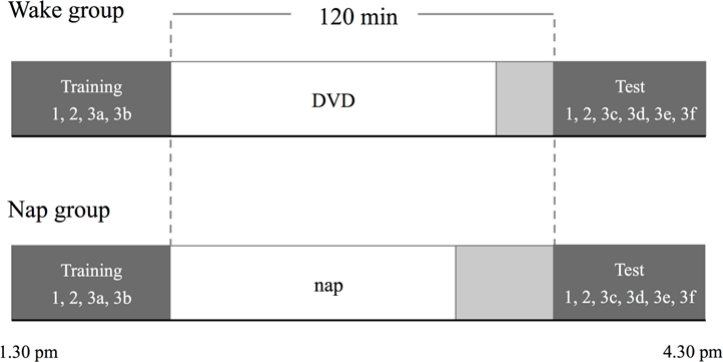
Experimental protocol for the two groups. The light grey area represents the break participants took before the test phase (nap = 30 min (to allow for the dissipation of sleep inertia); wake = 15 min). The numbers refer to different tasks: 1. declarative memory, 2. procedural memory, 3. language tasks (training: 3a: word repetition, 3b: word-picture matching; test: 3c: cued recall, 3d: determiner selection, 3e: word-picture matching with generalization sets, 3f: auditory translation recognition).

Participants were taught the novel words (e.g. *tib scoiffesh*) using a word-picture matching task [66], and after a 2-hour delay that included nap or wake their memory of the arbitrary and systematic components was tested. As well as recall and recognition tasks involving trained material, we also used a more stringent test of participants’ extraction of regularities involving generalization of the systematic mappings to untrained word-picture pairs. In addition to the language tasks, participants were also trained and tested on a declarative [15] and a procedural memory [67, 68] task (Fig 1).

## Method

### Participants

Fifty-one students at the University of York participated in the study after giving a written informed consent. They were monolingual native speakers of English, with no reported prior history of drug or alcohol abuse, neurological, psychiatric or sleep disorders. The data for five participants (one wake, four nap) were excluded from the analyses due to missing data for one or more tasks due to a programming error. There were 23 participants in each group in the analyses (nap: mean age = 19.6 years, 5 left handed, 12 female; wake: mean age = 20.0 years, 2 left handed, 17 female). Participants were required to abstain from alcoholic drinks and caffeinated products for 24 hours before the session and throughout the course of the study. The study protocol was approved by the Ethics Committee at the Department of Psychology, University of York.

### Materials

#### Language Training Set

The training language consisted of a total of 16 novel words. Each word consisted of a “stem” and a “suffix”, both pronounceable English pseudowords selected from the ARC database [69]. There were 16 unique stems. Each stem was combined with one of the four suffixes: *-eem*, *-esh*, *-ool*, *-aff*, with each suffix being part of four different words. Each resulting word (e.g. *bisesh*, *heefaff*) was assigned one of the two determiners (*tib*, *ked*) such that all words with the suffixes–*eem* or–*esh* were paired with the determiner *tib*, and all words with the suffixes–*aff* or–*ool* with the determiner *ked* (e.g. *tib bisesh*, *tib darleem*, *ked heefaff*, *ked jorool*).

The words were paired with pictures presenting stereotypically female or male occupations or characters (e.g. ballerina, cowboy). The pictures were selected from the commercially available ClipArt image database. To control for any unintended associations between specific word forms and pictures two versions of the training language were created: In Language 1 the pictures of female occupations and characters were presented with the novel words with the determiner *tib* and suffixes–*eem* or–*esh*, and the pictures of male characters or occupations were presented with the words with the determiner *ked* and suffixes–*ool* or–*aff* (see Table 1 for examples). In Language 2 the pictures of female occupations and characters were presented with the novel words with the determiner *ked* and suffixes–*ool* or–*aff*, and the pictures of male characters or occupations were presented with the novel words with the determiner *tib* and suffixes–*eem* or–*esh*. In both versions there was no phonological overlap between the English word for the depicted character/occupation and the novel word.

**Table 1:** Example items in different conditions in the training and generalization sets for Language 1. Feminine gender cues are marked in italics, masculine in bold.

	Determiner	Stem + Suffix	Picture
		*Training*	
feminine	*tib*	chush*eem*	*ballerina*
	*tib*	bis*esh*	*queen*
masculine	**ked**	jor**ool**	**pirate**
	**ked**	heef**aff**	**footballer**
		*Generalization*: *Determiner + Suffix*	
consistent	*tib*	zimb*esh*	*cheerleader*
	**ked**	mig**ool**	**king**
inconsistent	*tib*	curr*esh*	**policeman**
	**ked**	felch**ool**	*nun*
		*Generalization*: *Suffix Only*	
consistent	*tib*	sarb*esh*	*witch*
	**ked**	larsh**ool**	**boxer**
inconsistent	*tib*	sheg**ool**	*mermaid*
	**ked**	jit*esh*	**knight**

#### Generalization Sets

We developed two sets of additional items to test generalization of the systematic aspects of the training language (see Table 1 for examples).

In the *Determiner + Suffix* set, half of the items were consistent with the training set regarding the mapping between the determiner, the suffix, and the natural gender of the pictured character. For example, for Language 1 in consistent items previously unseen novel words with the determiner *tib* and the suffixes–*esh* or–*eem* were paired with pictures of previously unseen female occupations or characters. For the other half of the items, the determiner and the suffix remained consistent with each other, but were paired with pictured characters of the opposite gender. For example, previously unseen novel words with the determiner *tib* and ending–*esh* or -*eem* were paired with pictures of previously unseen male occupations or characters. There were 16 items (per language version) in this set, 8 per consistency condition, with an equal number of items with the two suffixes in each condition.

In the *Suffix Only* set, half of the items were again consistent exactly as described above, and the other half mismatched, but this time just on the suffix. For example, for Language 1 in inconsistent items previously unseen novel words with the determiner *tib* and suffix–*ool* or–*aff* were paired with pictures of previously unseen female characters. Here, the determiner is consistent with the female referent, but the suffix is not. Again there were 16 items (per language version) in this set, 8 per consistency condition, with an equal number of items with the two suffixes in each condition.

All words (both the training and the generalization sets) were digitally recorded in a sound-proof booth by an English native speaker. Two versions of each word in the training set were recorded, one with the determiner and one without it (e.g. *tib bisesh*, *bisesh*). The versions with the determiners were used at training (see below), and both versions (with and without the determiners) were used at test. The items with the determiners were recorded twice so that different recordings of the same item were used at training and at test.

#### Procedure

In brief, all participants were trained on a short declarative and a procedural memory task (described in S2 File) before they received the training on the language tasks. The training phase lasted approximately 35 minutes. Immediately subsequent to training the participants were informed about whether they were in the wake or the nap group. Participants in the nap group were taken to bed, and had a 90-minute sleep opportunity. Upon awakening, the nap participants had a 30-minute break to reduce the effects of sleep inertia, after which the test phase of the study started. The participants in the wake group watched a DVD with low verbal content for 105 minutes, and then took a 15-minute break before the start of the test phase. Thus both groups were tested after a 120 minute delay (see Fig 1).

Upon arrival at the lab the participants in the nap group were prepared for the polysomnographic recording using a standard EEG montage with 2 frontal, 2 central and 2 occipital electrodes referenced to the contralateral mastoid applied according to the 10–20 system. Two EOG and two EMG electrodes were also applied. The recording setup and the sleep staging analyses followed the American Academy of Sleep Science Manual [70].

Immediately before the start of the training and test phases of the study all participants filled out standard questionnaires assessing levels of sleepiness, motivation and concentration [71].

The training phase for all participants started at approximately 1.30 pm (Fig 1). The training tasks proceeded in the following order: 1. declarative memory training, 2. procedural memory training, 3. novel word learning—word repetition, 4. novel word learning—word-picture matching.

The test phase started 2 hours after the training phase. The tasks were administered in the following order: 1. declarative memory test, 2. procedural memory tests, 3. language tests: a. cued recall (trained set), b. determiner selection (trained set), c. word-picture matching with the generalization sets (the order of the two sets was counterbalanced across participants), d. auditory translation recognition (trained set). The test phase lasted approximately 30 minutes.

*Language training–Novel word repetition*: This task was used to familiarize participants with the phonological forms of the novel words to facilitate acquisition of word-meaning mappings at the following stage [72]. Each trial started with the presentation of a fixation cross at the center of the screen for 500 ms. A novel word with its determiner (e.g. *tib bisesh*) was then played over the headphones, and participants were asked to repeat it as accurately as possible. Each item was presented 3 times, for a total of 48 trials. The experimental items were preceded by two practice items.

*Language training–Word-picture matching*: The meaning of the novel words was taught using a word-picture matching task. This task was modeled on a well-established method of training meanings of novel words [66, 73], which involves presenting the correct target picture for a word in only a certain proportion of the trials (in this case, two thirds), while for the rest of the trials the word is presented with different randomly selected pictures from the training set. Participants are asked to decide whether the picture matches the word, but are not given feedback on their response, and so can only improve if they are sensitive to the consistencies across trials. This method was used in the current study to discourage the use of explicit strategies in learning the artificial language [66]. Each trial in this task started with the presentation of a fixation cross at the center of the screen for 500 ms, followed by a novel word and its determiner played over the headphones; 200 ms after the onset of the word a picture was displayed, which stayed on the screen for 1500 ms or until the participant pressed a response button, whichever came first. The participant’s task was to press a button on the response pad indicating whether they thought the word and the picture matched. They were encouraged not to think too much about the task but to respond “based on their intuition”. Participants were presented with each novel word a total of 12 times, 8 times paired with the target picture and 4 times with a pseudo-randomly selected non-target picture (twice with the picture with the same natural gender as the target picture, twice with a picture of the opposite gender). There were in total 192 trials, presented across 4 blocks. The match and mismatch trials were equally distributed across the four blocks.

*Language tests–Cued recall*: This task was used to assess memory of the trained words at test. Half of the trials included the target picture that had been associated with the novel word at training as the cue for recall, and half additionally included the initial grapheme (e.g. *b* for the picture of a queen (*bisesh)*). Each trial started with the presentation of a fixation cross for 500 ms, followed by the presentation of the target picture with or without the grapheme underneath. The participant’s task was to name the picture using the novel words they had learned earlier. No feedback was provided. The experimental items were preceded by a block of two practice items. The performance on the cued recall task was used to assess the memory of the arbitrary sound-meaning mappings (individual vocabulary items) by measuring accuracy of the recall of the stem portion of the novel words (*bis* in *bisesh*). This task was also used to assess the memory for the systematic aspects of the trained words by measuring accuracy of recall of the suffix portion of the novel word (*esh* in *bisesh*). In both cases, responses were coded as correct if all produced phonemes matched the target item. The separate analyses of the stem and the suffix portions of the word are a standard method of analyzing morphological aspects of word knowledge in psycholinguistics [74, 75].

*Language tests–Determiner selection*: This task was used to assess memory for the determiner associated with the pictured character in the trained items. Each trial started with the presentation of a fixation cross for 500 ms, which was followed by the presentation of a trained word without its determiner over the headphones (e.g. *bisesh*); 200 ms after the onset of the word the target picture was presented. Participants were asked to press a button on a response pad to indicate whether the word was a *tib* or a *ked* word. The determiners corresponding to the left and the right button on the response pad were printed in the corners of the screen throughout the trial. The left-right position of the buttons/determiners was counterbalanced across participants. The next trial was initiated automatically after a button press or 2500 ms after trial onset, whichever came first. No feedback was provided.

*Language tests—Word-picture matching with generalization sets*: This test was used to assess generalization of the systematic aspects of the learned mapping to new untrained items using the same word-picture matching task as at training. The items from the Determiner + Suffix, and the Suffix Only sets were presented in separate blocks, with the order of blocks counterbalanced across participants. Each new word-picture pair from the generalization sets was presented once. The participants’ task was to press a button on a response pad indicating whether they thought the word and the picture matched. As in training, they were encouraged not to think too much about the task but to respond “based on their intuition”. The proportion of match responses (endorsement rate) was used as a measure of performance on this task.

*Language tests—Auditory translation recognition*: This test was used to assess the memory for the meanings of the novel words. Each trial started with the presentation of the fixation cross for 500 ms, which was followed by the auditory presentation of an English word (e.g. queen) followed by 800 ms of silence, followed by a novel word (e.g. *bisesh*). All items were presented over the headphones. The English word matched the trained meaning of the novel word on half of the trials. For the non-matching trials, we used the English translations of the other novel words from the training set, such that the (non-matching) English word still matched the natural gender of the trained meaning of the novel word (e.g. the non-matching trial for *bisesh* was nurse). This ensured that the participants couldn’t use the natural gender of the referent of the English word to guess the meaning of the novel word. The participant’s task was to indicate whether the two words matched by pressing a button on a response pad. A non-parametric measure of discrimination, a’ [76], which takes into account the proportions of hits and false alarms was used as a measure of performance in this task.

For all analyses presented below we report Pearson’s *r* as a measure of effect size for the sleep manipulation, with *r* values of .10 indicating a small effect size, .30 a medium effect size and .50 a large effect size [77]. Degrees of freedom corrections were applied when the data did not meet the homogeneity of variance or sphericity assumptions [77]. The data from the declarative and procedural memory tasks were unrevealing and are presented in S2 File.

## Results

### Performance at training

**Fig 2:**
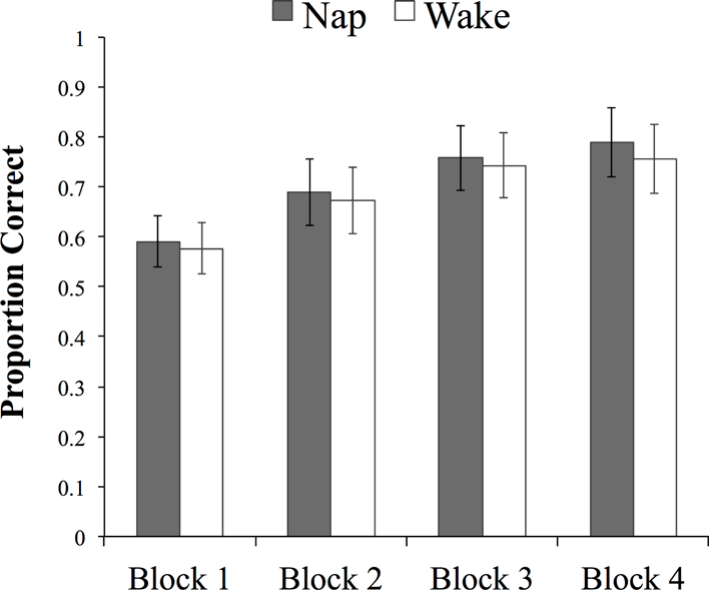
Accuracy (proportion of correct responses) during training in the two groups. Error bars represent 95% CIs.

Participants in the two groups learned the training set to the same degree: A mixed ANOVA with arcsine transformed proportion of correct responses in the word-picture matching task as the dependent variable, and group (nap vs. wake) as a between-subjects independent variable, and block (1 to 4) as a within-subjects independent variable, yielded no main effect of group (F(1, 44) = 0.57, p = .45; 95% CI of the difference [-.06, .13]). Participants in both groups improved across the four training blocks to the same degree (main effect of block: F(1.96, 86) = 46.76, p < .0001; no group x block interaction; see Fig 2 (accuracy in proportion correct presented for ease of interpretation)).

### Post-delay performance

#### Individual form-meaning mappings

**Fig 3:**
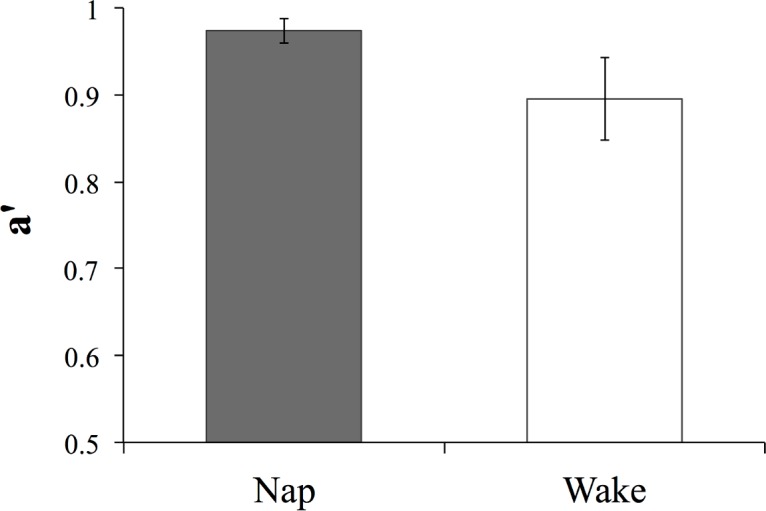
Performance on the auditory translation recognition task for the two groups. Error bars represent 95% CIs.

At test, both groups of participants showed good memory of the meanings of the novel words as measured by the auditory translation recognition task (average proportion of correctly recognized items: nap = .95, wake = .84). Crucially, participants who had been given an opportunity to nap showed better memory for the meanings of the novel words than the participants who had spent the same period awake. A *t*-test using a non-parametric measure of discrimination, a’, taking into account the proportions of hits and false alarms, confirmed a significantly better performance on the recognition task in the nap group relative to the wake group (t(24.93) = 3.05, p = .005; 95% CI of the difference [.03, .14]; *r*_*group*_ = .54; Fig 3).

**Fig 4:**
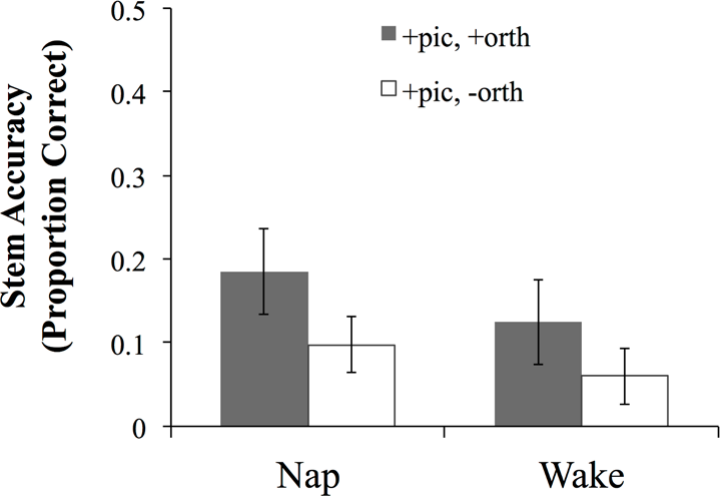
Proportion of correctly recalled stems in the cued recall task in the two groups for the conditions with and without the additional orthographic cue. Error bars represent 95% CIs.

We also tested participants’ memory of the novel words in a cued recall task. To derive a pure measure of the arbitrary component of this task, we categorized responses according to whether the stem component (e.g., *bis* in *bisesh*) of the response was correct, ignoring the suffix. The cue for recall in this task was the picture of the referent with which the word had been associated at training, or the picture plus the initial grapheme of the novel word. The overall recall accuracy of the stem portion of the word (proportion of correctly recalled stems) was low in both groups (M_nap_ = .15, M_wake_ = .08), but the nap group again outperformed the wake group (Fig 4). This was confirmed in a mixed ANOVA with arcsine transformed proportion of correctly produced stems as the dependent variable, and group and presence of the additional orthographic cue as independent variables, which yielded a main effect of group (F(1, 44) = 5.34, p = .026, 95% CI of the difference [.02, .23]; *r* = .33). Unsurprisingly, both groups recalled significantly more stems in the condition with the additional orthographic cue (cue type: F(1, 44) = 32.40, p < .0001; no group x cue type interaction).

Together these findings clearly indicate that, relative to an equivalent time spent awake, a 90-minute nap benefits the strengthening of the memory of the newly acquired arbitrary sound-meaning mappings reflected in the memory of individual words. This benefit is observed at quite different levels of performance, close to both ceiling (translation recognition) and floor (cued recall), and in both cases the effect size of the sleep manipulation was medium to large. This result provides further evidence in support of the role of sleep-associated consolidation in learning novel words, and specifically in the context of the arbitrary mapping between a novel phonological form and a meaning.

#### Determiners and suffixes: performance on trained items

We measured knowledge of the systematic aspects of the sound-meaning mappings using two types of tasks. First, for the trained items, we measured memory for the determiners using a determiner selection task, and for the suffixes using the cued recall task. Performance in these tasks could be based on individual item associations (e.g. recalling that the picture of the queen was associated with the determiner *tib*) or on more generalized knowledge (e.g. knowing that *tib* is associated with female pictures). Thus to the extent that the performance on the task reflects the memory of individual item associations some effects of sleep-related memory consolidation may be observed. Hence in the second series of tests we assessed the ability to generalize the systematic regularities to new previously unseen items. Here there is no memory for the specific picture-word associations, making it a purer test of extraction of systematic regularities.

**Fig 5:**
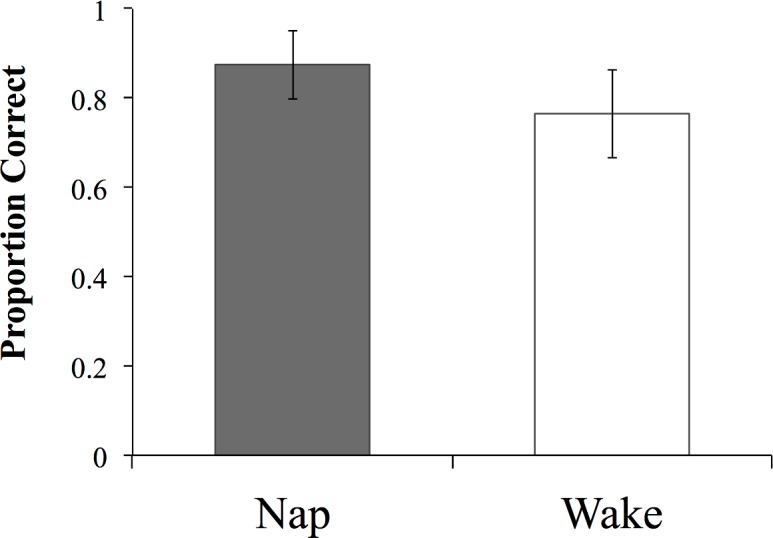
Accuracy (proportion of correct responses) on the determiner selection task in the two groups. Error bars represent 95% CIs.

*Determiner Selection*: Participants in both groups showed good memory for the determiners of the trained items (average proportion of correctly selected determiners: Nap = .88, Wake = .77; Fig 5). The performance in both groups was significantly better than chance (t_nap_ (22) = 10.1, p < .001, t_wake_ (22) = 5.52, p < .001), and it did not differ significantly between the groups (with arcsine transformed proportion of correct responses: t(44) = 1.81, p = .08, 95% CI of the difference [-.02, .36]; *r*_*group*_ = .26). Numerically, the nap group performed better than the wake group, however, as mentioned above, performance on this task is determined by both memory of individual arbitrary picture-word pairings and memory of systematic regularities. Thus the increase in the strength of individual arbitrary item-level mappings in the nap group found in the translation recognition and cued recall tasks could have contributed to their numerically better performance in this task.

**Fig 6:**
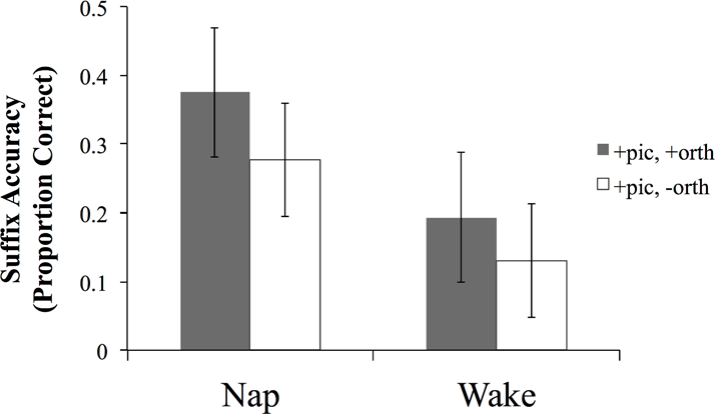
Proportion of correctly recalled suffixes in cued recall for the two groups for the conditions with and without the additional orthographic cue. Error bars represent 95% CIs.

*Suffixes*: We measured the knowledge of the suffixes for the trained items using accuracy of the recall of the suffix portion of the novel word (e.g. *esh* in *bisesh*) in the cued recall task. Overall accuracy of suffix recall was low in both groups (average proportion of correctly recalled suffixes: Nap = .33, Wake = .16), but the participants in the nap group recalled significantly more suffixes of the trained items than participants who had spent an equivalent time awake (Fig 6). An ANOVA with group and cue type as independent variables, and arcsine transformed proportion of correctly recalled suffixes as the dependent variable yielded a main effect of group (F(1, 44) = 9.68, p = .003; 95% CI of the difference [.09, .42]; *r*_*group*_ = .42). Participants in both groups recalled significantly more suffixes in the condition with the additional orthographic cue (cue type: F(1, 44) = 26.01, p < .0001; no group x cue interaction).

Similar to the results with word stems, these findings suggest that there is a sleep-related benefit for the memory of suffix-picture mappings for the trained items.

Thus the boost in the performance with the trained suffixes in the nap group could provide further evidence of the role of sleep in the strengthening of individual sound-meaning mappings (i.e., participants may treat the *esh* string as having been part of the sequence previously associated with the specific picture of a *queen*). Alternatively, this result could indicate that sleep-associated consolidation of the novel words aided the extraction of a grammatical gender regularity (e.g. feminine gender: *eem*, *esh* + female; masculine gender: *ool*, *aff* + male). To distinguish between these two alternatives, we performed additional analyses of the responses on the cued recall task.

We specifically focused on the cases where participants produced an incorrect response, but where this response included one of the suffixes from the training language (*eem*, *esh*, *aff*, or *ool;* all other responses were excluded from this analysis). The hypothesis was that if sleep-associated consolidation aided the abstraction of the suffix+natural gender regularity where, for example, the suffixes -*eem* and–*esh* are associated with female gender, an incorrect response for a picture of a trained female character should be more likely than chance to contain a ‘feminine’ suffix. Thus, for instance, when participants produce an incorrect response for the picture of a queen (correct novel word: *bis****esh***) it should be more likely to contain the gender-appropriate ending (*–eem*) than a gender inappropriate (masculine) ending (*–ool* or–*aff*). Chance performance for the gender-appropriate ending (.33) would suggest that the enhancement of the recall of the accurate suffixes in the nap over the wake group is more likely to be attributed to the superior memory of individual sound-meaning mappings rather than a suffix+natural gender regularity extraction.

This analysis included all incorrect responses where the produced ending was one of the phonologically ‘legal’ suffixes, i.e. one of the four suffixes of the trained language (-*esh*, -*eem*, -*aff*, -*ool*). Thus in both groups we only included participants whose responses included errors of this type (picture + orthography condition: n_nap_ = 15, n_wake_ = 10; picture only condition: n_nap_ = 13, n_wake_ = 10). All responses were further coded for whether the suffix was appropriate for the grammatical gender. For example, if the trained suffix for a picture was–*eem*, an incorrect but grammatical gender appropriate suffix would be–*esh*, while the gender inappropriate suffixes would be–*ool* and–*aff*. For each participant we calculated the proportion of incorrect but grammatically appropriate endings and compared it to chance (.33). If participants’ responses reflect the extraction of the grammatical gender schema, then they should be more likely than chance to contain grammatically appropriate suffixes. However, this was not the case: on average, approximately one third of incorrect phonologically legal endings were grammatically appropriate in both the nap and the wake group (Nap_pic+ orth_ = .31, Nap_pic only_ = .33; Wake_pic+ orth_ = .33, Wake_pic only_ = .40), and none were significantly different from chance (nap: t _pic+ orth_ (14) = -.24, p = .82; t_pic only_ (12) = .03, p = .98; wake: t _pic+ orth_ (9) = .03, p = .98; t_pic only_ (9) = .48, p = .64). These results suggest that the superior recall of suffixes for the nap participants reflects the strengthening of individual arbitrary sound-meaning mappings (e.g, (*bis)esh +* queen), rather than the extraction of a suffix + natural gender regularity (e.g., -*esh*, *-eem* + female). We tested this more directly in tasks assessing grammatical gender schema generalization to untrained items.

#### Determiners and suffixes: generalization

Generalization to novel items represents a stringent test of the abstraction of regularities. Thus to assess the extent to which participants’ performance on the systematic aspects of the trained language reflects sleep-associated benefits in the extraction of systematic regularities we used new untrained items in a word-picture matching task. We first present the results with the generalization set focusing on the regularity in the suffix-natural gender mapping (Suffix Only generalization set in Table 1).

Half of the items in this set were consistent regarding the suffix-natural gender regularity in the trained items (e.g. *eem*, *esh* + female), and half were inconsistent (e.g. *eem*, *esh* + male). In the inconsistent items the suffix was also inconsistent with the determiner (e.g. **ked** jit*esh*), however the determiner always matched the trained determiner-natural gender mapping (e.g. *ked* + male referent). If participants’ performance in the recall task reflects the extraction of the grammatical gender regularity rather than the strengthening of the sound-meaning mapping at the level of individual items then participants should be more likely to endorse the consistent than the inconsistent items.

**Fig 7:**
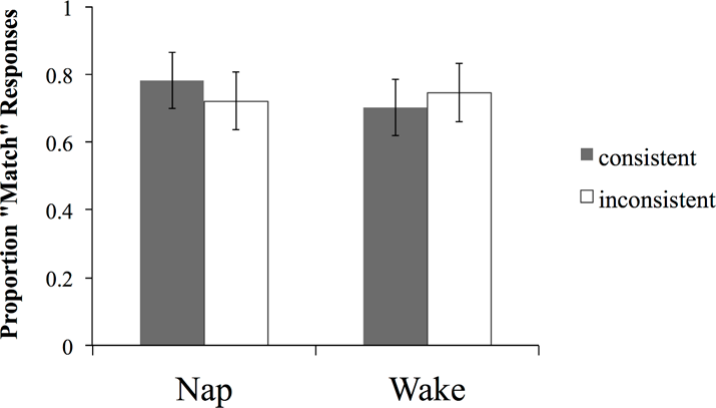
Endorsement rate (proportion of ‘match’ responses) on the word-picture matching task with the Suffix Only generalization set for the two groups. Error bars represent 95% CIs.

However, this pattern was not found (Fig 7). A mixed ANOVA with group (nap vs. wake) and consistency with the trained grammatical regularities (consistent vs. inconsistent) as independent variables, and endorsement rate (proportion of match responses) as the dependent variable yielded no significant main effects or interactions (main effects: all Fs < 1; group x consistency interaction: F(1, 44) = 3.02, p = .09; *r*_group x consistency_ = .25; this trend was marginal partly due to an unexpected and likely spurious numerically higher endorsement rate for *inconsistent* items in the wake group).

These findings, showing insensitivity to consistency suggest that participants in neither group abstracted and generalized the grammatical regularity (suffix + natural gender) of the trained items to the new untrained items. These results provide convergent evidence with the findings from the recall task, in that they suggest that the nap-related superior recall of the novel word suffixes reflects the strengthening of the arbitrary form-meaning mapping for individual vocabulary items rather than the extraction of the systematic grammatical gender regularity. Thus we did not see evidence that sleep-related consolidation aided the abstraction of a grammatical gender schema as it is reflected in the suffix to gender mappings.

It is important to note that both groups endorsed the items regardless of consistency with the suffix-natural gender regularity at the above chance levels (wake: t_consistent_(22) = 4.82, p < .001; t_inconsistent_(22) = 6.49, p < .0001; nap: t_consistent_(22) = 7.08, p < .001; t_inconsistent_(22) = 4.7, p < .001). We hypothesized that this preference for a “match” response in both conditions indicated that the participants’ performance in this task was driven by the fact that the mapping between the determiner and the natural gender of the new word-picture pairs was consistent with the training set in both conditions in this generalization set. In other words, participants were possibly making use of the determiner cue to gender, which always matched, but showed no evidence of making use of the more complex suffix cue. Further evidence consistent with this hypothesis is provided by the findings from the Determiner + Suffix generalization set.

**Fig 8:**
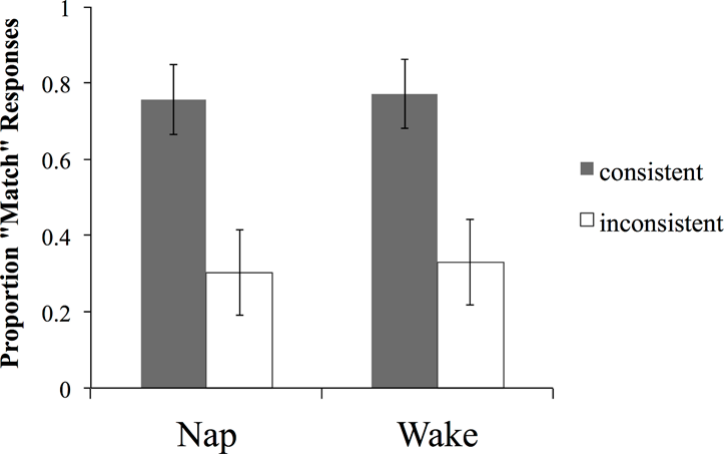
Endorsement rates (proportion of ‘match’ responses) on the word-picture matching task with untrained items for the two groups, for the Determiner + Suffix generalization set. Error bars represent 95% CIs.

As in the Suffix Only generalization set, the Determiner + Suffix generalization set contained items consistent or inconsistent with the grammatical regularities in the training set (consistent: *tib* + *esh/eem* + female; *ked* + *ool/aff* + male; inconsistent: *tib* + *esh/eem* + male; *ked* + *ool/aff* + female; see Table 1 for more examples). A mixed ANOVA with group (nap vs. wake) and consistency with the regularity in the training set as independent variables, and endorsement rate as the dependent variable, yielded a main effect of consistency (F(1, 44) = 50.13, p < .0001) with a higher endorsement rate for the items consistent with the trained regularity (Fig 8). There was no main effect of group, or group x consistency interaction (both Fs < 1; *r*_*group x consistency*_ = .01). Both groups of participants were also significantly more likely than chance to endorse the items with the mapping consistent with the trained regularities (nap: t(22) = 5.71, p < .001; wake: t(22) = 5.93, p < .0001) and reject the ones inconsistent with the trained regularities (nap: t(22) = -3.4, p = .003; wake: t(22) = -3.23, p = .004). These findings indicate that the participants in both groups extracted this systematic form-meaning regularity in the training set and generalized it to new untrained items. Together, these findings suggest that a systematic grammatical regularity involving a salient cue (determiners) can be abstracted relatively easily after a short exposure, and successfully generalized to previously unseen items. We found no evidence that sleep is necessary or beneficial for this process.

#### Relationship with sleep stages

Nap participants spent on average 74.7 minutes asleep. Approximately a third of this time (Table 2) was spent in slow wave sleep (SWS, stages 3 and 4). The proportion of time spent in SWS did not correlate with the memory of individual items as assessed by auditory translation recognition (*r* = .01, p = .98), stem (*r* = .18, p = .41) or suffix recall (*r* = .09, p = .70). The proportion of time spent in SWS did not correlate with the performance on the generalization tasks (items consistent with the trained regularities: Determiner + Suffix *r =* .16, p = .47; Suffix only *r* = .32, p = .14; items inconsistent with the trained regularities: Determiners + Suffix *r* = -.19; p = .39; Suffix only *r* = .08, p = .71).

**Table 2:** Average duration of sleep stages during the nap.

	Percentage of Total Sleep Time	Minutes
Stage 1	17.7%	12.26
Stage 2	44.5%	33.65
SWS (stages 3 and 4)	32.5%	24.52
REM	5.8%	4.67

#### Sleep questionnaires

The difference in fatigue/alertness between the two groups was assessed using the Stanford Sleepiness Scale (SSS). We also assessed self-reported tiredness, drowsiness, motivation and concentration. There were no significant differences between the two groups on any of the scales at the start of the training phase. At the start of the test phase participants in the wake group had significantly higher scores on the Stanford Sleepiness Scale (M_wake_ = 3.52, M_nap_ = 1.86, t(42) = -5.37, p < .001), and they reported being more tired (M_wake_ = 3.52, M_nap_ = 2.13, t(44) = -4.33, p < .001), more drowsy (M_wake_ = 3.30, M_nap_ = 1.87, t(44) = -3.87, p < .001), and less concentrated (M_wake_ = 4.17, M_nap_ = 5.48, t(44) = 3,64, p = .001) and less motivated (M_wake_ = 4.13, M_nap_ = 5.30, t(44) = 3.61, p = .001). In addition, for the participants in the nap group there was a significant negative relationship between the proportion of time spent in Stage 2 sleep and SSS scores, and self-reported tiredness and drowsiness (Table 3). This suggests that properties of sleep contributed to the group differences on these measures, in addition to any differences in fatigue/alertness.

**Table 3:** Beta coefficients in the multiple regressions with SSS, tiredness and drowsiness at the beginning of the test phase and the proportion of time spent in Stage 2 and SWS.

	Stage 2	SWS
SSS	-.574**	.076
tiredness	-.500*	-.157
drowsiness	-.657**	.073

**p < .001

*p < .05

To assess the contribution of the differences in fatigue/alertness between the nap and the wake group to the performance on the tasks, we ran analyses of covariance using SSS scores as a covariate. The results of these analyses (S1 File) confirm the benefits of a 90-minute nap for the strengthening of the memory of arbitrary information, measured by the translation recognition task. These benefits do not extend to the systematic form-meaning mappings reflected in determiners and suffixes.

## Discussion

We explored the role of sleep-related memory consolidation in learning novel form-meaning mappings in an artificial language. Our study focused on the dimension of systematicity/arbitrariness of the mapping, with some aspects of the language being arbitrary and others being more systematic. We hypothesized that the involvement of sleep-related consolidation would vary such that the strongest contribution would be reflected in the consolidation of the arbitrary mappings (e.g. between word stems and referents), and the weakest contribution in the consolidation of systematic mappings (e.g. between determiners/suffixes and the natural gender of the referents). This is indeed what we found: participants’ memory for individual vocabulary items was improved after a 90-minute daytime nap compared with the same time spent awake. This effect was found in both a translation matching task and a cued recall task. For cued recall it is possible that greater fatigue for the wake participants played a role in the sleep-wake difference, but such an alternative does not hold for translation matching.

There were several tests of the systematic aspects of the same artificial language. Some of these tests assessed memory of the trained items and showed improved memory for the nap relative to the wake group. In particular, the suffix components of the trained items were recalled better after sleep relative to an equivalent time awake. Does this result suggest that sleep aids systematicity? We think not. If sleep aided the abstraction of the systematic mappings, we would expect them to be generalized in that the incorrect responses would also be more likely to reflect the systematic suffix-gender regularity in the nap but not the wake group. However, the analysis of recall errors did not show any sensitivity to the systematic mapping between the suffix and the natural gender of the referent in either group. Thus this finding suggests that the better performance on suffix recall in the nap group reflects improved memory for the individual arbitrary form-meaning mappings, rather than the extraction of the systematic regularity.

In contrast to the tests of the memory of the trained items, the two generalization tests showed no sleep benefit for application of systematic knowledge. The key property of these tests is that they use previously unseen items and so are less likely to rely on the memory of the trained arbitrary item pairings. These of course were null effects, but the fact that there were several tests that varied greatly in their difficulty and nature helps to strengthen the conclusion that sleep was not influential. Indeed performance on the determiners and suffixes was quite different: On the one hand, participants in both sleep and wake groups showed good evidence of having acquired the knowledge of the link between the determiners and the natural gender of the referents. On the other hand, there was no evidence that either group had picked up the relevance of the more subtle suffix cues–possibly because of the contribution of several factors, including the more complex nature of the mapping, the temporal organization of the redundant cues [56, 57], the noise from the mismatch trials in the training task, or the decreased variability of the stems (e.g. [78], but cf. [79–81]). Crucially, we found no evidence that sleep helped the extraction of this form-meaning regularity. Importantly, while the generalization tests showed null effects for the sleep/wake manipulation, they both showed significant effects for the linguistic (consistency) manipulations. Thus it is unlikely that the tests themselves did not have enough sensitivity to detect differences in performance.

Together these findings provide evidence in support of the Complementary Learning Systems model [25] in that learning of the arbitrary form-meaning mappings thought to depend on the hippocampal system benefited from the sleep-associated memory consolidation. This was unlike the systematic aspects of the new form-meaning mappings which were either acquired well but not additionally benefitted by sleep in the case of the salient determiner cue, or were learned as part of the individual form-meaning mappings for words but not as a systematic grammatical regularity, for the less perceptually salient and more complex suffix cue.

**Fig 9:**
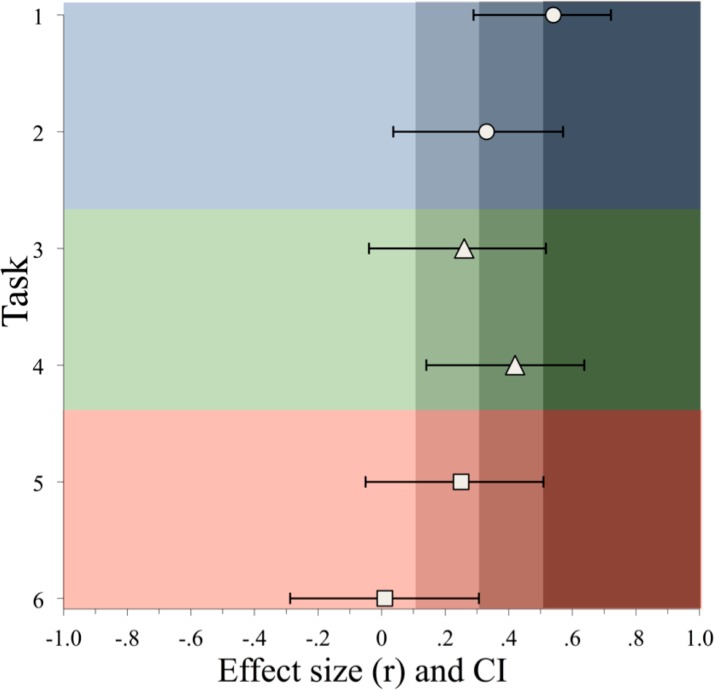
Effect size (*r*) with 95% CIs [84] for tasks measuring the memory for arbitrary and systematic aspects of the mappings for trained (tasks 1–4) and new items (tasks 5 and 6). Circles: arbitrary mappings (top to bottom: auditory translation recognition, cued recall). Triangles: arbitrary + systematic mappings in trained items (top to bottom: determiner selection, suffix recall). Squares: systematic mappings in new items (top to bottom: suffix only generalization, determiner + suffix generalization). The shaded areas indicate small (r = .10), medium (r = .30) and large (r = .50) effect sizes

The fact that the same learning episodes in the current study consisted of *both* arbitrary *and* systematic components, mutually competing for long-term consolidation, is possibly crucial. It is exactly under these conditions where sleep is suggested to be selective or preferential in its involvement [82]. The complexity of the new memories may have contributed to the preferential involvement of sleep in prioritizing the most hippocampally reliant components over the multi-item generalizations that can also be supported by the neocortical system [25, 82]. Thus, we are not arguing that sleep cannot facilitate the more systematic components of new memories [83], but we are suggesting that when learning involves complex multi-componential new memories typical of real word learning this would happen perhaps over the longer term, or when arbitrary components are de-emphasized or better established in memory. This suggestion is supported in the current study by the graded effect sizes for the sleep manipulation, with stronger effects for the clearly arbitrary tests, weaker effects for the tests that could rely on either arbitrary or systematic knowledge, and then weakest effects for the purer tests of systematic knowledge (Fig 9).

The existing literature on the role of sleep-related consolidation in regularity extraction and generalization is not straightforward. Some studies have reported sleep benefits in regularity extraction where the regularities involved sequential dependencies between tones [85, 86], letters [87] or syllables [2, 7]. However, these studies differ from the current one in important ways. The encoding of sequential dependencies may be chiefly reliant on procedural memory systems [88, 89], unlike the cross-modal form-meaning mappings used in the current study. Further, the new memories created in those studies involved fewer components to be consolidated in that they involved sequential dependencies that related to a single type of stimulus (e.g. tones, letters or syllables), unlike the current study, and word learning in general, which involves at least two different stimulus types (i.e. word forms and their meanings), and multiple regularities within and across the two stimulus types. It is exactly this additional complexity which may have brought about the more selective role of sleep in consolidation processes [82]. Additional differences between the current study and studies reporting sleep benefits in schema abstraction are related to the differences in the populations tested: In the studies by Gomez and colleagues [2, 7] sleep benefits in the extraction of sequential dependencies were found in 15-month old infants, whose sleep patterns are significantly different from adult sleep patterns [90]. Thus the extent to which these findings are generalizable to adult populations is less clear (see [91] for further discussion).

As mentioned, our study was intended to pit against each other the arbitrary and systematic aspects of a new declarative memory. Previous studies have tended to focus on one or other aspect, but one exception is a recent nap study in a non-linguistic domain [92]. Here the authors trained participants on face-location associations in which there was either a systematic mapping between a set of facial features and screen locations or there was no systematic association between the two (i.e. the face-location mapping was arbitrary). The systematically related face-location pairs were initially learned better than the arbitrarily related ones, and they were also forgotten to a lesser degree over time. Crucially, and as in the current study, generalization tests assessing the change in the memory for the systematically related aspects did not show any sleep-specific improvements. Thus, when pitted against each other in the same stimulus set, systematic aspects of new memories in a non-linguistic domain seem not to show preferential benefits related to sleep-associated consolidation, similar to the findings in the current study.

Recent studies using stimulus types more similar to ours [64, 93, 94] point to two additional dimensions to consider: the extent to which the test tasks require rapid access to the newly learned information, and the relationship between the memory of individual items and the memory of regularities that are found across a set of items. For example, participants in the Tamminen et al. [93] study learned meanings of novel affixes attached to existing words (e.g.–*nule* in *sleepnule*, meaning a participant in a sleep study, or *climbnule*, meaning a climber of mountains with dangerous peaks). The participants were able to generalize the knowledge of the meanings of the novel affixes in a non-speeded task immediately after learning, but in a speeded task the generalization was only present at a delayed test two days after training. Thus, similar to the current study which used non-speeded tasks, Tamminen and colleagues found evidence of generalization of the systematic regularities immediately after learning, and the benefits of consolidation were only seen in tasks which required rapid access to the newly learned information (see also [30, 65]). Similarly, in a nap paradigm using a stimulus set pairing novel prefixes with existing words, Batterink et al. [94] reported fast learning of the systematic regularities at training which was not further influenced by sleep. However, a behavioral index of the use of the systematic regularities in a speeded task after a nap was associated with the interaction between SWS and REM. Interestingly, in the Tamminen et al. [93] study there was evidence of a *decline* in recognition memory for the trained items over time (see also [65]). Similar findings were reported in a study by Lau et al. [64]: the memory for trained items was worse while simultaneously there was improved generalization of regularities for the participants who napped relative to the participants who stayed awake. Thus in both of these studies there was evidence of a trade-off between generalization performance and trained item memory.

This set of findings is related to a hypothesis suggested by Werchan and Gomez [95] that forgetting of (irrelevant) properties of individual items may be a crucial dimension in the process of regularity extraction: When learning a set of new mappings with both arbitrary and shared, systematic, components, the stronger memory of the individual arbitrary components may interfere with the extraction of the regularity in the shared components. Conversely, the weakening of the memory of the arbitrary, irrelevant components may facilitate the abstraction of the systematic, shared components. Following this logic, in the case of word learning as in the current study, where successful performance on the task prioritizes the arbitrary sound-meaning mappings, the strengthening of the arbitrary components during sleep may have interfered with the generalization based on the more systematic elements, which are less relevant for learning the meaning of the new word (see [92] for a related argument). Both Werchan and Gomez [95] and Lau et al. [64] have shown a pattern of loss of arbitrary components promoting better generalization, but these studies differ in the extent to which sleep and wake are associated with these changes. In the current study we see evidence of the same trade-off but from the opposite point of view, where stronger memory of arbitrary form-meaning mappings may have hindered benefits for the extraction of regularities.

In sum, improved generalization of new knowledge may depend on a complex interplay between the memory of the arbitrary and the systematic components of new mappings, with the nature and complexity of the generalization dictating the relative benefits of sleep and wake. Although the basic prediction of greater dependency of the arbitrary components of a novel mapping on sleep can be easily derived from a complementary systems account, the further complexities would require detailed simulations, incorporating a more explicit account of wakeful forgetting and a better consideration of the changes in consolidation processes as a mapping becomes more familiar and better established in memory. Speculatively, it is feasible that a complementary systems model when learning a mapping over repeated sleep/wake cycles would gradually shift in terms of the nature of the hippocampal involvement. Hippocampal replay during sleep would initially lead to better learning of the most arbitrary aspects of a mapping in the neocortex, meaning that these aspects would rely less on hippocampal mediation when the same set of mappings are next encountered during wake. With less reliance on the hippocampal structures for the arbitrary aspects of the mapping, the next cycle of sleep associated replay would then further strengthen the more systematic aspects of the mapping. This kind of characterization of the sleep-wake process is similar to Lewis and Durrant’s [83] model of schema formation during sleep, but emphasizes the relevance of the systematicity/arbitrariness dimension as a regulatory factor in the learning and consolidation process.

One further point of comparison between our study and previous sleep studies is worth mentioning. Two studies have examined the extraction of hierarchical structure from pairs of associated items. Ellenbogen and colleagues [16] used a transitive inference paradigm, in which participants learned arbitrary preference choices between “premise pairs” (e.g., A>B, B>C, C>D, D>E, E>F). After an interval of sleep or wake, they were tested for their memory of these arbitrary relationships and also of their knowledge of the (untrained) hierarchy. The groups had comparable levels of performance on the trained pairs, but the sleep group showed a superior performance on the test of the implicit hierarchy (i.e., B>E). Similar results were observed by Coutanche and colleagues [96] using a two-dimensional spatial hierarchy. Both these studies, then, seem to be in opposition to the current one in that they show a benefit of sleep not for arbitrary item knowledge but instead for the more abstract structure.

However, there are two aspects of these studies that may help to explain their rather different results. First, in both studies, the memory for the trained pairs was numerically better for the sleep group than the wake group, although this difference of about 4–5% did not reach significance level in either case. Thus it could be that small improvements in the memory for all the individual items led to improvements also in the ability to connect these elements to perform relational judgments (cf. [26]). Second, unlike the current experiment, both the above studies used reinforcement learning to facilitate learning of the premise pairs. This aspect is likely to be important: Werchan and Gomez [97] replicated Ellenbogen et al. [16] again using reinforcement learning, but found that the sleep advantage for hierarchical knowledge disappeared when the training task involved observational learning of the premise pairs. Speculatively, the reinforcement aspect of training may have favored striatal systems of learning, lessening the involvement of the hippocampus [98]. The task we used in the current experiment is more like the observational learning of Werchan and Gomez [97], and was intended to recruit the hippocampus for initial learning [99]. Therefore, it may be that quite detailed aspects of learning can affect the neural systems that are recruited in learning, and that sleep may affect their consolidation in different ways. Although we do not wish to argue that all aspects of language learning utilize a single memory system, it is certainly important to understand the properties of the hippocampal learning system that clearly has a central role to play in language learning.

## Conclusions

Our findings demonstrate that when people encounter complex, multi-componential mappings for the first time we see evidence of a prioritization process in the sleep-associated consolidation of declarative memory: sleep is particularly beneficial for the long-term retention of the arbitrary form-meaning mappings typically found when learning the meanings of new words. In contrast, we found no evidence for involvement of sleep in learning the systematic aspects of the mapping. In these circumstances, the role of sleep in regularity extraction may only become evident when arbitrary information has been consolidated. Such a pattern of prioritization fits with the predictions of a complementary systems model of learning, in which reliance on the hippocampal component of the model is greater for arbitrary as opposed to systematic mappings.

## Supporting Information

S1 File ANCOVA Results for Language Tasks. (PDF)

S2 File Declarative and Procedural Memory Tasks. (PDF)
