# Supplementary material for: Does Sleep Improve Your Grammar? Preferential Consolidation of Arbitrary Components of New Linguistic Knowledge
Source: PLoS One. 2016 Apr 5;11(4):e0152489. doi: 10.1371/journal.pone.0152489 (PMC4821602; doi:10.1371/journal.pone.0152489)
Supplement: S1 File — (PDF) [file pone.0152489.s001.pdf]

S1: ANCOVA Results for Language Tasks

In order to assess the contribution of the differences in the measures of fatigue/alertness between the nap and the wake group to the performance on the language tasks at test, we performed analyses of covariance using the scores on the Stanford Sleepiness Scale (SSS) as a covariate. The SSS scores for two participants in the nap group were missing, and thus they were excluded from the analyses.

Post-delay performance: Arbitrary sound-meaning mappings

For the auditory translation recognition task, which measures the memory for the arbitrary sound-meaning mappings (vocabulary), we ran the ANCOVA with group (nap vs. wake) as an independent variable, and *a'* as the dependent variable. This analysis yielded a main effect of group, with the nap group outperforming the wake group ($F(1, 41) = 8.54$, $p < .01$; 95% CI of the difference [.03, .18]; $r = .42$), and no significant main effect or interactions with the covariate (main effect: $F(1, 41) = .99$, $p = .33$).

We also measured the memory of the novel vocabulary in a recall task, in the accuracy of the recall of the stem portion of the novel words (e.g. *bis* in *bisesh*), which was low in both groups ($M_{nap}= .15$, $M_{wake} = .08$). The ANCOVA was run with group and type of cue (picture only vs. picture + first grapheme) as a between and a within-subjects independent variable respectively, and arcsine transformed proportion of correctly recalled stems as the dependent variable. The analysis yielded no significant main effects or interactions (group: $F(1, 41) = 1.63$, $p = .21$; 95% CI of the mean difference [-.05, .24]; $r = .20$), and no significant main effect or interactions with the covariate (main effect: $F (1, 41) = .57$, $p = .46$).

These findings indicate that the improved performance on the recall test measuring vocabulary knowledge in the nap group could partially be attributed to the differences in the measures of fatigues/alertness between the two groups. Importantly, though, the sleep benefit in the nap group remains for the memory of individual arbitrary sound-meaning

mappings as measured by the recognition task when measures of fatigue/alertness have been taken into account.

Post-delay performance: Systematic sound-meaning mappings

We tested the memory of the suffixes in the trained items using accuracy of the recall of the suffix portion of the novel words (e.g. *esh* in *bisesh*) in the cued recall task. The ANCOVA with group and type of cue as independent variables, and arcsine transformed recall accuracy of the suffixes yielded a significant main effect of cue ($F(1, 41) = 6.47$, $p = .015$) with a better recall with the additional presence of the orthographic cue. No other main effects or interactions were significant (group: $F(1, 41) = 1.68$, $p = .20$; 95% CI for the mean difference [-.08, .36], $r = .20$) , and there was no significant main effect or interactions with the covariate (main effect: $F (1, 41) = 3.26$, $p = .08$).

For the determiner memory in the trained items as measured by the determiner selection task, the ANCOVA with group as the independent variable and arcsine transformed accuracy yielded no main effect of group ($F(1, 41) = 1.3$, $p = .26$; 95% CI for the difference [-.11, .40]; $r = .18$), and no main effect or interactions with the covariate (main effect: $F(1, 41) = .08$, $p = .78$).

We assessed the performance with the systematic mapping involving the suffix in a generalization test with a new set of previously unseen items (the Suffix Only set). Half of the new word-picture pairs were consistent with the mapping between the ending and the determiner and the natural gender of the referent present in the training set, and half were inconsistent (see Table 1 for examples). The ANCOVA was run with group (nap vs. wake) and consistency as independent variables, and endorsement rate of the new word-picture pairs as the dependent variable. This analysis yielded no significant main effects or interactions (group: $F(1, 41) = .61$, $p = .44$; 95% CI for the mean difference [-.09, .19]; $r = .12$), and no significant effect of the covariate ($F(1, 44) = .07$, $p = .79$).

For the generalization test assessing the systematic sound-meaning mapping including the determiner and the suffix (the Determiner + Suffix set) the ANCOVA with group and consistency as the independent variables, and endorsement rate as the dependent variable, yielded a marginally significant effect of consistency $F(1, 41) = 3.65$, $p = .063$; 95% CI of the difference [.33, .60]), and no other main effects or interactions (group: $F(1, 41) = .08$, $p = .78$; 95% CI of the difference [-.10, .08]; $r = .04$). The covariate did not yield a significant main effect ($F(1, 41) = .03$, $p = .88$), and it did not interact with other factors.

These findings provide further evidence of the lack of sleep benefit for the memory of the systematic aspects of the novel form-meaning mappings.
