# Supplementary material for: Does Sleep Improve Your Grammar? Preferential Consolidation of Arbitrary Components of New Linguistic Knowledge
Source: PLoS One. 2016 Apr 5;11(4):e0152489. doi: 10.1371/journal.pone.0152489 (PMC4821602; doi:10.1371/journal.pone.0152489)
Supplement: S2 File — (PDF) [file pone.0152489.s002.pdf]

S2: Declarative and Procedural Memory Tasks

We provide below the description and the analyses of the performance on the tasks measuring declarative and procedural memory.

Method

Declarative memory: A 2D object location task was used as a declarative memory task (Rasch, Buechel, Gais, & Born, 2007). The task consisted of 15 card pairs of familiar objects and animals presented on a 6 x 5 grid on a computer screen. At training, participants were first introduced to the cards using a simple exposure task, where each trial started with the presentation of the first card in the pair for 1s, followed by the presentation of the second card for 3s. The inter-trial interval was 3s. (All non-target cards were presented as gray squares on the grid.) Participants were presented with each pair twice. Their task was to try to remember the location of the cards for a later test. The second phase of training consisted of a cued recall task, where the first card was presented on the screen and the participant's task was to click on the screen location where they thought the second card of the pair may be. Visual feedback was presented after each trial for 2s, regardless of accuracy. Each pair was presented three times during this phase. Accuracy on the third block of trials in the cued recall was used as the measure of declarative memory performance at training. The same cued recall task was used at test, with a single presentation of each pair.

Procedural memory*:* A finger tapping task (Walker, Brakefield, Morgan, Hobson, & Stickgold, 2002; Walker et al., 2003) was used as a procedural memory task. In each trial in this task a 5-element sequence of digits (e.g. 4-1-3-2-4) was displayed on a computer screen for 30 s. The participant's task was to tap the sequence on a computer keyboard (using the keys Z, X, C, V, which were labeled as numbers 1, 2, 3, 4) using the four fingers of his/her non-dominant hand. At training, the sequence was presented for 12 trials, with the inter-trial interval of 30 s. The number of correct sequences tapped for each 30s interval for the last four trials was used as the measure of performance at training. Participants were randomly assigned one of the two sequences of digits (4-1-3-

2-4, 2-3-1-4-2). At test, participants were first presented with the trained (old) sequence for 4 trials, which was followed by a new sequence (the sequence used for the counterbalancing at training) for 4 trials. The performance on the new sequence was used as a control for nonspecific changes in motor performance (Wilhelm, Diekelmann, & Born, 2008). The same measure of performance was used at test as at training.

Results

Declarative memory: The performance on the 2D object location task was analyzed using an ANCOVA with group (nap vs. wake) and test (train vs. test) as independent variables, SSS scores as a covariate, and accuracy as the dependent variable. The analysis yielded no significant main effects or interactions (Figure 1; test: $F(1, 41) = 1.23$, $p = .27$; group: $F(1, 41) = 1.35$, $p = .25$; test x group: $F(1, 41) = .08$, $p = .77$). There was also no significant effect of the covariate ($F(1, 41) = .09$, $p = .77$). This indicates that participants' performance on the declarative memory task did not change over a 2-hour delay period spent asleep or awake (Figure 1).

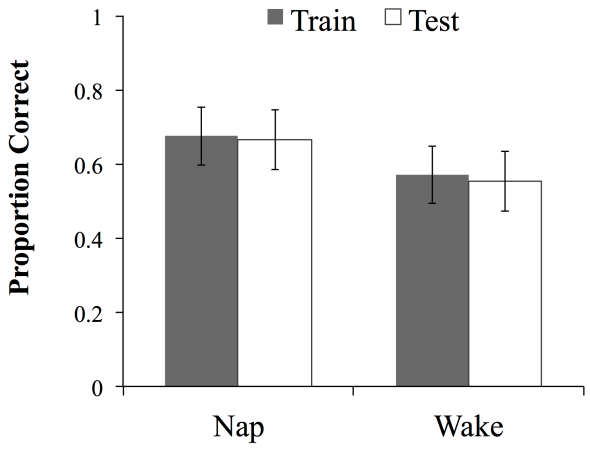

Figure 1. Accuracy (proportion of correctly selected cued locations) in the 2D object location task. Error bars represent 95% CIs.

Procedural memory: The data from three participants from the nap group were not recorded for the procedural task due to a technical error. For the remainder of the

participants, the ANCOVA with group (nap vs. wake) and test (train vs. test) as independent variables, SSS scores as a covariate, and accuracy (number of correct sequences per trial) for the old sequence as the dependent variable yielded a significant main effect of group ($F(1, 39) = 4.77$, $p = .035$) with the overall better performance on the task in the nap group (Figure 2), and no main effect of test ($F(1, 39) = 1.75$, $p = .19$). There was a numerically larger change in the performance between training and test in the nap group relative to the wake group, but the group x test interaction did not reach significance ($F(1, 39) = 3.25$, $p = .079$). The covariate did not yield a significant effect ($F(1, 39) = 1.18$, $p = .28$).

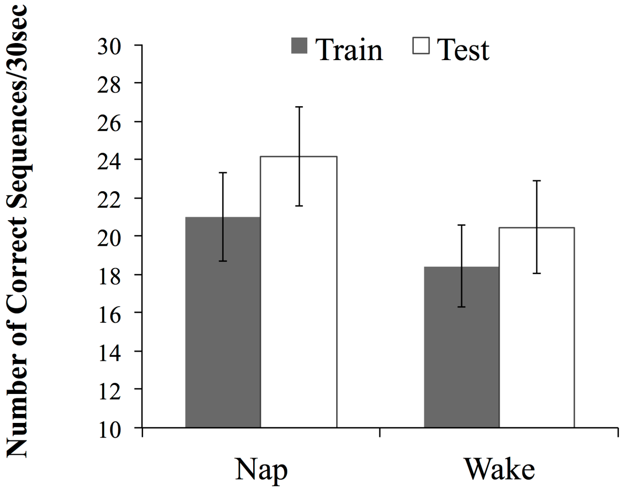

Figure 2. Accuracy (number of correct sequences per trial) on the finger tapping task for the two groups. Error bars represent 95% CIs

For each participant we also compared the difference in performance at test relative to training for the old vs. the new sequence. A significant change in performance for the old but not for the new sequence would indicate that the offline gains reflect sequence-specific rather than general changes in motor performance (Wilhelm et al. 2008). Participants in both groups showed an overall improvement only in the performance on the old sequence ($M_{nap} = 3.41$, $M_{wake} = 2.03$). The performance with the new sequence was poorer relative to the training performance with the old sequence ($M_{nap} = -2.66$, $M_{wake} = -2.75$). The ANOVA with the performance change as the dependent variable

yielded a main effect of type of sequence ($F(1, 40) = 141.45$, $p < .0001$), and no main effect of group or group x sequence interaction. These findings provide some evidence of sequences-specific, but not sleep specific, offline gains in procedural memory as measured by the finger tapping task.

In sum, the findings from the declarative and procedural memory tasks indicate that in the current study, which included a series of memory tasks at training, we do not see a benefit of a 90-minute nap for the declarative and procedural memory consolidation measured by a spatial and a finger tapping task. These findings are consistent with evidence that memory systems interact during off-line consolidation and that consolidation in one system can block the consolidation in the other (Brown & Robertson, 2007).

References:

Brown, R. M., & Robertson, E. M. (2007). Off-line processing: reciprocal interactions between declarative and procedural memories. *Journal of Neuroscience, 27*, 10468-10475.

Rasch, B., Buechel, C., Gais, S., & Born, J. (2007). Odor cues during slow-wave sleep prompt declarative memory consolidation. *Science, 315*, 1426-1429.

Walker, M. P., Brakefield, T., Morgan, A., Hobson, J. A., & Stickgold, R. (2002). Practice with sleep makes perfect: Sleep-dependent motor skill learning. *Neuron, 35*, 205-211.

Walker, M. P., Brakefield, T., Seidman, J., Morgan, A., Hobson, J. A., & Stickgold, R. (2003). Sleep and the time course of motor skill learning. *Learning & Memory, 10*, 275-284.

Wilhelm, I., Diekelmann, S., & Born, J. (2008). Sleep in children improves memory performance on declarative but not procedural tasks. *Learning & Memory, 15*, 373-377.
